# Supplementary material for: Protocol for a scoping review to understand what is known about how GPs make decisions with, for and on behalf of patients who lack capacity
Source: BMJ Open. 2020 Oct 20;10(10):e038032. doi: 10.1136/bmjopen-2020-038032 (PMC7577062; doi:10.1136/bmjopen-2020-038032)
Supplement: Author's manuscript [file bmjopen-2020-038032.draft_revisions.pdf]

BMJ Open

BMJ Open is committed to open peer review. As part of this commitment we make the peer review history of every article we publish publicly available.

When an article is published we post the peer reviewers' comments and the authors' responses online. We also post the versions of the paper that were used during peer review. These are the versions that the peer review comments apply to.

The versions of the paper that follow are the versions that were submitted during the peer review process. They are not the versions of record or the final published versions. They should not be cited or distributed as the published version of this manuscript.

BMJ Open is an open access journal and the full, final, typeset and author-corrected version of record of the manuscript is available on our site with no access controls, subscription charges or pay-per-view fees (http://bmjopen.bmj.com).

If you have any questions on BMJ Open's open peer review process please email info.bmjopen@bmj.com

BMJ Open

Protocol for a scoping review to understand what is known about how GPs make decisions with, for and on behalf of patients who lack capacity

Journal:	*BMJ Open*
Manuscript ID	bmjopen-2020-038032
Article Type:	Protocol
Date Submitted by the Author:	26-Feb-2020
Complete List of Authors:	Ogden, Simon; University of Bristol, Centre for Ethics in Medicine<br>Ives, Jonathan; University of Bristol, Centre for Ethics in Medicine<br>Huxtable, Richard; University of Bristol, School of Social and Community Medicine
Keywords:	PRIMARY CARE, MEDICAL ETHICS, QUALITATIVE RESEARCH, STATISTICS & RESEARCH METHODS

SCHOLARONE™
Manuscripts

I, the Submitting Author has the right to grant and does grant on behalf of all authors of the Work (as defined in the below author licence), an exclusive licence and/or a non-exclusive licence for contributions from authors who are: i) UK Crown employees; ii) where BMJ has agreed a CC-BY licence shall apply, and/or iii) in accordance with the terms applicable for US Federal Government officers or employees acting as part of their official duties; on a worldwide, perpetual, irrevocable, royalty-free basis to BMJ Publishing Group Ltd ("BMJ") its licensees and where the relevant Journal is co-owned by BMJ to the co-owners of the Journal, to publish the Work in this journal and any other BMJ products and to exploit all rights, as set out in our licence.

The Submitting Author accepts and understands that any supply made under these terms is made by BMJ to the Submitting Author unless you are acting as an employee on behalf of your employer or a postgraduate student of an affiliated institution which is paying any applicable article publishing charge ("APC") for Open Access articles. Where the Submitting Author wishes to make the Work available on an Open Access basis (and intends to pay the relevant APC), the terms of reuse of such Open Access shall be governed by a Creative Commons licence – details of these licences and which Creative Commons licence will apply to this Work are set out in our licence referred to above.

Other than as permitted in any relevant BMJ Author's Self Archiving Policies, I confirm this Work has not been accepted for publication elsewhere, is not being considered for publication elsewhere and does not duplicate material already published. I confirm all authors consent to publication of this Work and authorise the granting of this licence.

For peer review only

Protocol for a scoping review to understand what is known about how GPs make decisions with, for and on behalf of patients who lack capacity.

Simon Jack Ogden (Correspondence author)

jack.ogden@bristol.ac.uk

Centre for Ethics in Medicine, Population Health Sciences, Bristol Medical School, Canynge Hall, 39 Whatley Road, Bristol, BS8 1PS, UK

Jonathan Ives

j.ives@bristol.ac.uk

Centre for Ethics in Medicine, Population Health Sciences, Bristol Medical School, Canynge Hall, 39 Whatley Road, Bristol, BS8 1PS, UK

Richard Huxtable

r.huxtable@bristol.ac.uk

Centre for Ethics in Medicine, Population Health Sciences, Bristol Medical School, Canynge Hall, 39 Whatley Road, Bristol, BS8 1PS, UK

Keywords: Best interests, Incapacity, Decision-making, Empirical bioethics

For peer review only

ABSTRACT

Introduction

GPs are regularly required to make decisions with, for and on behalf of patients who lack capacity. In England these decisions are made for incapacitated adult patients under the Mental Capacity Act 2005 (MCA),[1] which primarily requires that decisions are made in the patient's "best interests".[2] Regarding children, decisions are also made in their best interests but are done so under the Children Act 1989, which places paramount importance on the welfare of the child. Decisions for children are usually made by parents, but a GP may become involved if they feel a parent is not acting in the best interests of the child. Internationally, including elsewhere in the UK, different approaches are taken.[3] We hypothesise that, despite the legislation and professional guidelines,[4] there are many different approaches taken by GPs in England when making these complex decisions with, for and on behalf of patients who lack capacity. To better understand what is known about how these decisions are made, we plan to undertake a scoping review and directed content analysis of the literature.

Methods and analysis

To ensure a wide breadth of literature is captured, a scoping review will be undertaken as described by Arksey and O'Malley (2005).[5] A five-stage approach will be taken when conducting this review: (1) Identifying the research question; (2) Identifying relevant papers; (3) Study selection; (4) Data extraction; and (5) Summarising and Synthesis. The final stage will include a directed content analysis of the data to help establish the cross-cutting themes.

Ethics and dissemination

The scoping review will be disseminated through conferences and peer-reviewed publications. This scoping review is the first (mapping) [6] phase in a proposed larger study to explore how GPs make decisions with, for and on behalf of those who lack capacity. Qualitative research with GPs, patients and their families will follow, before all the results are synthesised using an "empirical bioethics" methodology.[7]

STRENGTHS AND LIMITATIONS OF THIS STUDY

- This scoping review will map the current understanding of how GPs make decisions with, for and on behalf of patients who lack capacity.
- Arksey and O'Malley's[5] scoping review methodology will be used and it will follow the PRISMA Extension for Scoping Reviews (PRISMA-ScR) checklist.[8]
- While the study will focus on GPs in England an international search will allow comparison of approaches between different legal jurisdictions.
- The data will be critically synthesised with reference to ethical and legal principles to identify cross-cutting themes and to ascertain where further research is needed on this increasingly important topic.

INTRODUCTION

GPs in the UK National Health Service provide over 300 million consultations per year[9] and the workload continues to rise year-on-year.[10,11] It has been said that the NHS is a victim of its own success; with novel and improved treatments, patients live longer, albeit with higher rates of co-morbidity and medical complexity, often with reduced capacity to make their own medical decisions.[12] As a result, GPs are seeing an increasing number of patients who are unable to make their own decisions about their medical treatment.[13] Some common conditions that often result

For peer review only

in decisions being made on behalf of the patient include dementia, learning disabilities, acquired traumatic brain injuries and mental health conditions.

In England the Mental Capacity Act 2005 (MCA) provides a legal framework for making decisions for patients who are unable to do so themselves. The MCA primarily states that if a patient is unable to make a particular decision, then it can be made for them in their "best interests" and must be done in a way that is least restrictive of their rights. Although the Act does not provide a formula for best interests decision-making, the Act does disclose some of the factors that must be considered when making these best interest decisions, including the patient's past and present wishes and feelings, the beliefs and values that would likely influence their decision if they had capacity and other factors that they would be likely to consider if they were able to do so. There is also a duty to consult with those close to the patient, such as their family, when making best interests decisions.

The MCA also empowers patients to make provision for a future in which they may lack the capacity to make decisions. Two options are available. First, the MCA provides for proxy decision-making by introducing "lasting power of attorneys", who can make decisions on behalf of patients when they lose capacity. However, these proxies must ultimately still conform to the rules set out in the MCA with regards to acting in the patient's best interests. Second, the MCA places advance decision-making on a statutory footing, by introducing the "advance decision to refuse treatment", which may refuse (but not consent to or demand) specified treatments that might otherwise be provided after the patient has lost capacity.

English law regarding the capacity to consent and refuse treatment in children is slightly more complicated. The treatment of children is generally governed by the Children Act 1989 (CA), which requires that the paramount consideration is the welfare, or "best interests", of the child. The Family Law Reform Act 1969 states that those aged 16 and 17 years of age have the same legal ability to consent as an adult, however this does not mean they have a right to refuse treatment (Re W (A Minor) [14) and indeed someone with parental responsibility (or a court) may consent on their behalf. In children younger than 16, the law surrounding their capacity to consent is set out in Gillick v West Norfolk and Wisbech AHA.[15] The term "Gillick competence" refers to a child under 16 who "reaches a sufficient understanding and intelligence to be capable of making up his own mind on the matter requiring decision".[16] As with older children, even a Gillick competent minor cannot expect his refusal of treatment to operate as a veto. As such, where a child refuses to consent, or lacks the competence to consent, decisions may be made by others, in the "best interests" of the child.

Internationally, different legal jurisdictions provide different approaches to making decisions with, for and on behalf of patients who lack capacity to make these decisions themselves. For example, in Scotland, patients are treated under the Adults with Incapacity (Scotland) Act 2000 where a doctor is required to complete a 'section 47' certificate if a patient is deemed to lack capacity and cannot use force unless immediately necessary. Conspicuous is the absence of the term "best interests" in the Adults with Incapacity (Scotland), instead it chooses to set out five principles which must be applied when making decisions. Any action or decision must benefit the person, it must be the least-restrictive option, take account of the wishes of the person, include a consultation with a relevant other and encourage the person to use existing skills and develop new skills. In Northern Ireland, patients are treated under the Mental Capacity (Northern Ireland) Act 2016 which covers the treatment of all patients who lack capacity, including those in whom incapacity arises as a result of a mental health condition. This is different to England, where there is separate legislation – the Mental Health Act 2007 – governing the treatment of those with mental disorders. It is, however,

For peer review only

similar in that it uses the 'best interests' test to guide how these decisions should be made. In the US decisions may be made by a nominated surrogate decision-maker, such as a family member.[17]

The United Nations Convention on the Rights of Persons with Disabilities (CRPD) is also of importance given that it was ratified by the United Kingdom in 2009 and is hailed by some as advocating for a supported decision making approach [18] rather than a substituted or surrogate decision making approach of which best interest decisions are an example. The UN Committee on the Rights of Persons with Disabilities stated that 'perceived or actual deficits in mental capacity must not be used as justification for denying legal capacity.'[19] It is explicitly stated by this UN committee that best interests decisions are substituted decisions and should be replaced by supported decisions. Returning to England, the law on best interest decisions has been further criticised as being vague, making its application open to divergent interpretations.[20] Neither Act – the MCA or the CA – *elaborates* a particular account of what is in a patient's best interests; rather, a non-exhaustive list of different factors is *enumerated*.[21,22] Different stakeholders and proxy decision-makers – such as clinicians, those with lasting powers of attorney, or parents – might point to different factors and reach different decisions, even in seemingly similar cases. We hypothesise that, despite the legislation and professional guidelines,[4,23,24] there are many different approaches taken by GPs when making these complex decisions depending on their interpretation of the facts, their own moral judgement, and their own understanding of the patient in front of them, with some (but not all) of whom they might have a longstanding relationship.

STUDY RATIONALE

With the increasing incidence of patients [13] who lack capacity to make decisions themselves comes an increasing need to understand how these decisions are made by GPs and how this process might be improved.

GPs have a unique role in the healthcare of patients and are often involved in the care of both the patient and their family, which can sometimes be for many years, from childhood to adulthood. GPs are also responsible for coordinating a patient's secondary care and managing most of their chronic diseases in the community setting.[25] While continuity of care has suffered in recent years, there is still a continuity in the relationship between GP and patient, which makes GPs well-placed to be involved in many of the complex medical decisions for patients who lack capacity.[26] The decisions being made could range from what treatment the patient should receive at the end of their life to whether a patient should be admitted to hospital.[27] Given these are often important decisions that have a significant impact both the patient and their family, it is important to understand how and why these decisions are made.

STUDY OBJECTIVES

The objective of this scoping review is to identify and map current research on how decisions are made with, for and on behalf of patients who lack capacity in primary care. The main concepts, approaches and theories will be established and synthesised with reference to current ethical principles and the various legal positions internationally. The overall objective is to identify knowledge gaps and areas where further research is needed.

METHODS AND ANALYSIS

Protocol design

A scoping review will be performed as described by Arksey and O'Malley [5] and set out in the PRISMA guidelines by Tricco et al.[8] A scoping review methodology has been chosen as it will allow

For peer review only

us to identify what is known about the key characteristics and factors that are important to decisions made with, for and on behalf of patients who lack capacity. Following initial searches, it was clear that there is limited research currently published in this area, making a scoping review an appropriate approach. Scoping reviews map the topic as a whole and highlight where gaps in knowledge exist, thus helping guide further research.[28] The aim is to understand the breadth and depth of existing knowledge.[29]

According to Arksey and O'Malley, there are six stages to undertaking a scoping review: identify the research question; identify relevant studies; study selection; data collection; summarising; and finally, expert consultation. The first five of these stages will be applied in this scoping review. The final consultation stage will be omitted as this will form part of the next phase of the research.

Stage 1: Identify research question

The research question was formulated in consultation with the research team and with consideration given to the further planned "empirical bioethics" research. "Empirical bioethics" combines theoretical and empirical research and may take various forms, but here will involve mapping what is currently known through literature reviews (this phase), empirical research to deepen our understanding, then shaping work, to recommend ways forward.[6] The question posed for the current mapping phase was intentionally kept broad in its wording so as to capture all the published research that speaks to this question, including both empirical and theoretical research. The main research question is:

- What is understood about how decisions are or should be made in primary care with, for and on behalf of patients who lack capacity?

Subordinate research questions include:

1. What approaches and processes are used in making decisions in primary care with, for and on behalf of patients who lack capacity?
2. Who participates in, or is consulted about, decisions in primary care with, for and on behalf of patients who lack capacity?
3. Which factors are considered by the participants when making decisions in primary care with, for and on behalf of patients who lack capacity?
4. What does the theoretical literature reveal about how decisions are or should be made in primary care with, for and on behalf of patients who lack capacity?

Stage 2: Identify relevant studies – search strategy

A limited initial search was carried out using MEDLINE (PubMed) and Web of Science to discover frequently used search terms, abbreviations, keywords and MeSH terms. This stage is important so that terms that might be used in different countries and disciplines are discovered and included in the final search string to capture all relevant research. A subject librarian also advised on the proposed search terms and approach.

Pilot searches were performed to test various search strings using different combinations of keywords in addition to wildcards (*) and Boolean modifiers to produce the final search string. The aim of the pilot searches was to capture as much of the relevant research as possible.

The article title and abstracts of papers will be searched in MEDLINE, Embase, PsychINFO, The Cochrane Library, Web of Science and JSTOR using the terms in the final search string:

[gp OR general pract* OR primary care] AND

For peer review only

[capacity or compet* or incapacit*] AND
[best interest* or decision making or decision]

Stage 3: Study selection

The title and abstracts of all the papers returned from the search will be transferred to the 6 software where they will be screened by the lead reviewer (SJO) according to the inclusion and exclusion criteria below. Papers will be classified into “include”, “exclude” and “unsure” groups. Duplicated papers will be removed.

A second reviewer (RH) will screen a sample of the included and excluded papers to aid reliability. The second reviewer will also review all those papers in the “unsure” group. Any disagreements that cannot be resolved by discussion will be resolved by bringing in a third reviewer (JI). The team will meet regularly to discuss results and will seek to reach consensus on the papers to include and exclude. Where consensus is not reached, a majority decision will be carried (i.e. include or exclude).

Once the list of included papers is agreed, the first reviewer will screen the references of included papers to check for further papers that might fit the inclusion criteria. Any additional papers found will repeat the process above, as needed.

All papers that are found to fit the inclusion and exclusion criteria will be included in the scoping review.

Inclusion

- Papers, books, book chapters, national guidelines.
- All types of empirical research including qualitative, quantitative and mixed methods.
- Theoretical studies.
- Any paper that addresses the question of *how* medical decisions are made for patients who lack capacity and *what* factors are considered important. It is not necessary for this to be the paper’s primary aim.
- Papers situated in the primary healthcare setting.
- Papers that consider decisions about healthcare treatment/non-treatment.
- Papers where decisions are made following advance care planning when patients were considered to have capacity.
- Any papers published since 1960 and in English.

Exclusion

- Grey literature including journalism.
- Papers that do not refer to lack of capacity or, in the case of a child, competence when referring to any medical decision making.
- Papers not situated in the primary healthcare setting.
- Papers that do not consider decisions about healthcare treatment/non-treatment e.g. that concern research and/or social care exclusively.
  Papers not in English.

Stage 4: Data extraction and analysis

A directed content analysis will be undertaken in order to identify and describe key themes present in the papers. A directed content analysis begins with a pre-established coding framework, but then allows new codes to be used, or existing codes to be further specified, as the analysis progresses.[30]

For peer review only

The first stage will be data extraction (or 'charting the results') - an iterative process that involves extracting predefined data into a charting sheet, which can then be used to explore themes. The draft charting table comprises key details about the papers, but also include the initial coding framework that will be applied to papers - focussing on 4 areas of results (see vii below). A description of what those papers say about each of those areas will be entered into the data extraction sheet, and then further sub codes will be developed, as appropriate, to further distinguish between, and record, different kinds of content.

i. Articles details
ii. Country of origin
iii. Broad category (i.e. patient situation/condition)
iv. Brief summary
v. Aims of study
vi. Methods/Type of Study
vii. Results
    a. Approaches and processes used in making decisions
    b. Participants involved in making decisions
    c. Factors considered in making decisions
    d. Theoretical argument(s) about how to make decisions
viii. Conclusion
ix. Reflections from reviewer

The first reviewer (SJO) will extract data from all included empirical and theoretical research into the charting table. Data from quantitative research will be described and charted. To improve reliability, 10-15% of papers will also have data extracted independently by a second (RH) and third (JI) reviewer.

The extracted data and expanded coding will be discussed amongst the group early in the data extraction stage to discuss disagreements and agree on how the coding framework should be developed and applied.

Stage 5: Summarising and synthesis

The second phase in the analysis will be to systematically explore the codes developed (and data extracted) in stage 4 to develop larger descriptive themes which summarise what the evidence from the papers show.

Any quantitative evidence will be simply described and reported narratively.

Finally, consideration will be given to what gaps in the literature exist and how these might inform further research on the topic.

PATIENT AND PUBLIC INVOLVEMENT

No patients involved.

ETHICS AND DISSEMINATION

One paper conveying the results of the review will be prepared for publication in a peer reviewed journal. The authors will also seek to present the findings at appropriate bioethics or primary care conferences in the UK. The results from this review will help guide the next phase of the research involving a qualitative "empirical bioethics" study, looking further into how GP's do and should make these decisions.

For peer review only

No ethics approval is required for this scoping review.

REFERENCES

1. The Mental Capacity Act 2005
2. s.4 The Mental Capacity Act 2005
3. DeMartino ES, Dudzinski DM, Doyle CK, et al. Who Decides When a Patient Can't? Statutes on Alternate Decision Makers. *N Engl J Med* 2017;376(15):1478–1482. doi:10.1056/NEJMms1611497
4. GMC Good medical practice (2013). General Medical Council http://www.gmc-uk.org/guidance/good_medical_practice.asp.
5. Arksey H, O'Malley L. Scoping studies: Towards a Methodological Framework. *Int J Soc Res* 2005;8:1,19-32. doi:10.1080/1364557032000119616
6. Huxtable, R., Ives, J. Mapping, framing, shaping: a framework for empirical bioethics research projects. *BMC Med Ethics* 2019;20,86. doi:10.1186/s12910-019-0428-0
7. Ives, Dunn, Cribb. Empirical Bioethics: Theoretical and Practical Perspectives. Cambridge University Press 2016.
8. Tricco AC, et al. PRISMA Extension for Scoping Reviews (PRISMA-ScR): Checklist and Explanation. *Ann Intern Med* 2018 Oct 2;169(7):467-473. doi:10.7326/M18-0850
9. NSH Five Year Forward View. 2014 https://www.england.nhs.uk/five-year-forward-view/next-steps-on-the-nhs-five-year-forward-view/primary-care/. Accessed December 2019
10. Hobbs FDR, et al. Clinical workload in UK primary care: a retrospective analysis of 100 million consultations in England. *Lancet* 2016 Jun 4;387(10035):2323-2330. doi:10.1016/S0140-6736(16)00620-6
11. Lay-Flurrie S, Mathieu E, Bankhead C, et al. Patient consultation rate and clinical and NHS outcomes: a cross-sectional analysis of English primary care data from 2.7 million patients in 238 practices. *BMC Health Serv Res* 2019;19(1):219. doi:10.1186/s12913-019-4036-y
12. Wallace E, Salisbury C, Guthrie B, et al. Managing patients with multimorbidity in primary care *BMJ* 2015; 350 :h176. doi:10.1136/bmj.h176
13. Wancata J, Musalek M, Alexandrowicz R, et al. Number of dementia sufferers in Europe between the years 2000 and 2050. *Eur Psychiatry* 2003;18:306–13.
14. Re W (Minors) [2016] EWHC 2226 (Fam).
15. Gillick v West Norfolk & Wisbeck Area Health Authority [1986] AC 112 House of Lords.
16. [1986] AC 112 at page 189.
17. Torke AM, Alexander GC, & Lantos J. Substituted Judgment: The Limitations of Autonomy in Surrogate Decision Making. *J Gen Intern Med* 2008; 23:1514–1517. doi:10.1007/s11606-008-0688-8
18. Kohn N, Blumenthal JA, Campbell AT. Supported Decision-Making: A Viable Alternative to Guardianship?. *Penn State Law Review* 2013;117:4 1111-1157 at 1129
19. UN Committee on the Rights of Persons with Disabilities. General comment no. 1, article 12: Equal recognition before the law, CRPD/C/GC/1 http://daccessdds-ny.un.org/doc/UNDOC/GEN/G14/031/20/PDF/G1403120.pdf
20. DeMartino ES, Dudzinski DM, Doyle CK, et al. Who Decides When a Patient Can't? Statutes on Alternate Decision Makers. *N Engl J Med* 2017;376(15):1478–1482. doi:10.1056/NEJMms1611497
21. Martin W, Freyenhagen F, Hall E, et al. An unblinkered view of best interests. *BMJ* 2012;345 :e8007. doi: 10.1136/bmj.e8007

For peer review only

22. Lim CM, Dunn MC, Chin JJ. Clarifying the best interests standard: the elaborative and enumerative strategies in public policy-making. *J Med Ethics* 2016;42:542–54. doi:10.1136/medethics-2016-103454
23. Best interests decision-making for adults who lack capacity A toolkit for doctors working in England and Wales. BMA.
24. General Medical Council: 0–18 years: guidance for all doctors. www.gmc-uk.org/ethicalguidance
25. Bodenheimer T, Wagner EH, Grumbach K. Improving primary care for patients with chronic illness: the chronic care model, part 2. *JAMA* 2002; 288: 1909–14. doi:10.1001/jama.288.15.1909
26. Campbell S M, Hann M, Hacker J, et al. Identifying predictors of high quality care in English general practice: observational study. *BMJ* 2001;323:784. doi:10.1136/bmj.323.7316.784
27. Tammes, P, Payne, RA, Salisbury, C, et al. The impact of a named GP scheme on continuity of care and emergency hospital admission: A cohort study among older patients in England, 2012-2016. *BMJ Open* 2019;vol.9,no.9,e029103. doi:10.1136/bmjopen-2019-029103
28. Peters, MDJ, Godfrey, C, Kahlil, H, et al. Guidance for conducting systematic scoping reviews. *Int J Evid Based Healthc* 2015;vol.13,ed.3,pp.141-46. doi:10.1097/XEB.0000000000000050
29. Levac D, Colquhoun H, O'Brien KK. Scoping studies: advancing the methodology. *Implement Sci* 2010;5(1):1. doi: 10.1186/1748-5908-5-69
30. Hsieh HS, & Shannon S. Three Approaches to Qualitative Content Analysis. *Qualitative health research* 2005;15. 1277-88. doi:10.1177/1049732305276687

AUTHORS' CONTRIBUTIONS

SJO conceived the idea for the scoping review. SJO, JI and RH all contributed to development of the research questions and study design. SJO drafted the initial protocol with input from JI and RH. All authors contributed to re-drafting and subsequent final revision of this protocol. SJO is the guarantor of the review.

ACKNOWLEDGEMENTS

I would like to express my appreciation to Dr Giles Birchley, Dr Zuzana Deans and Dr Emanuele Valenti and Dr Iona Sherwood for the general advise, help and support they have given in conceiving this project.

FUNDING STATEMENT

This work was supported by the Wellcome Trust Institutional Strategic Support Fund, grant number 204813/Z/16/Z.

COMPETING INTEREST STATEMENT

We have read and understood the BMJ policy on declaration of interests and declare that we have no competing interests.

WORD COUNT: 2719

For peer review only

Reporting checklist for protocol of a systematic review.

Based on the PRISMA-P guidelines.

Instructions to authors

Complete this checklist by entering the page numbers from your manuscript where readers will find each of the items listed below.

Your article may not currently address all the items on the checklist. Please modify your text to include the missing information. If you are certain that an item does not apply, please write "n/a" and provide a short explanation.

Upload your completed checklist as an extra file when you submit to a journal.

In your methods section, say that you used the PRISMA-Preporting guidelines, and cite them as:

Moher D, Shamseer L, Clarke M, Ghersi D, Liberati A, Petticrew M, Shekelle P, Stewart LA. Preferred Reporting Items for Systematic Review and Meta-Analysis Protocols (PRISMA-P) 2015 statement. Syst Rev. 2015;4(1):1.

		Reporting Item	Page Number
**Title**			
Identification	#1a	Identify the report as a protocol of a systematic review	1
Update	#1b	If the protocol is for an update of a previous systematic review, identify as such	n/a
**Registration**			
	#2	If registered, provide the name of the registry (such as PROSPERO) and registration number	n/a
**Authors**			
Contact	#3a	Provide name, institutional affiliation, e-mail address of all protocol authors; provide physical mailing address of corresponding author	1
Contribution	#3b	Describe contributions of protocol authors and identify the guarantor of the review	9
**Amendments**			

For peer review only

	#4	If the protocol represents an amendment of a previously completed or published protocol, identify as such and list changes; otherwise, state plan for documenting important protocol amendments	n/a
**Support**			
Sources	#5a	Indicate sources of financial or other support for the review	9
Sponsor	#5b	Provide name for the review funder and / or sponsor	9
Role of sponsor or funder	#5c	Describe roles of funder(s), sponsor(s), and / or institution(s), if any, in developing the protocol	n/a
**Introduction**			
Rationale	#6	Describe the rationale for the review in the context of what is already known	4
Objectives	#7	Provide an explicit statement of the question(s) the review will address with reference to participants, interventions, comparators, and outcomes (PICO)	4
**Methods**			
Eligibility criteria	#8	Specify the study characteristics (such as PICO, study design, setting, time frame) and report characteristics (such as years considered, language, publication status) to be used as criteria for eligibility for the review	5
Information sources	#9	Describe all intended information sources (such as electronic databases, contact with study authors, trial registers or other grey literature sources) with planned dates of coverage	6
Search strategy	#10	Present draft of search strategy to be used for at least one electronic database, including planned limits, such that it could be repeated	5
Study records - data management	#11a	Describe the mechanism(s) that will be used to manage records and data throughout the review	7
Study records - selection process	#11b	State the process that will be used for selecting studies (such as two independent reviewers) through each phase of the review (that is, screening, eligibility and inclusion in meta-analysis)	6
Study records - data collection process	#11c	Describe planned method of extracting data from reports (such as piloting forms, done independently, in duplicate), any processes for	7

For peer review only

		obtaining and confirming data from investigators	
Data items	#12	List and define all variables for which data will be sought (such as PICO items, funding sources), any pre-planned data assumptions and simplifications	6
Outcomes and prioritization	#13	List and define all outcomes for which data will be sought, including prioritization of main and additional outcomes, with rationale	7
Risk of bias in individual studies	#14	Describe anticipated methods for assessing risk of bias of individual studies, including whether this will be done at the outcome or study level, or both; state how this information will be used in data synthesis	7
Data synthesis	#15a	Describe criteria under which study data will be quantitatively synthesised	7
Data synthesis	#15b	If data are appropriate for quantitative synthesis, describe planned summary measures, methods of handling data and methods of combining data from studies, including any planned exploration of consistency (such as I2, Kendall's τ)	7
Data synthesis	#15c	Describe any proposed additional analyses (such as sensitivity or subgroup analyses, meta-regression)	7
Data synthesis	#15d	If quantitative synthesis is not appropriate, describe the type of summary planned	n/a
Meta-bias(es)	#16	Specify any planned assessment of meta-bias(es) (such as publication bias across studies, selective reporting within studies)	n/a
Confidence in cumulative evidence	#17	Describe how the strength of the body of evidence will be assessed (such as GRADE)	n/a

The PRISMA-P checklist is distributed under the terms of the Creative Commons Attribution License CC-BY 4.0. This checklist was completed on 25. February 2020 using https://www.goodreports.org/, a tool made by the EQUATOR Network in collaboration with Penelope.ai

For peer review only

BMJ Open

Protocol for a scoping review to understand what is known about how GPs make decisions with, for and on behalf of patients who lack capacity

Journal:	*BMJ Open*
Manuscript ID	bmjopen-2020-038032.R1
Article Type:	Protocol
Date Submitted by the Author:	26-Jul-2020
Complete List of Authors:	Ogden, Simon; University of Bristol, Centre for Ethics in Medicine<br>Huxtable, Richard; University of Bristol, School of Social and Community Medicine<br>Ives, Jonathan; University of Bristol, Centre for Ethics in Medicine
**Primary Subject Heading**:	General practice / Family practice
Secondary Subject Heading:	Ethics, Mental health, Paediatrics, Patient-centred medicine, Research methods
Keywords:	PRIMARY CARE, MEDICAL ETHICS, QUALITATIVE RESEARCH, STATISTICS & RESEARCH METHODS

SCHOLARONE™
Manuscripts

I, the Submitting Author has the right to grant and does grant on behalf of all authors of the Work (as defined in the below author licence), an exclusive licence and/or a non-exclusive licence for contributions from authors who are: i) UK Crown employees; ii) where BMJ has agreed a CC-BY licence shall apply, and/or iii) in accordance with the terms applicable for US Federal Government officers or employees acting as part of their official duties; on a worldwide, perpetual, irrevocable, royalty-free basis to BMJ Publishing Group Ltd ("BMJ") its licensees and where the relevant Journal is co-owned by BMJ to the co-owners of the Journal, to publish the Work in this journal and any other BMJ products and to exploit all rights, as set out in our licence.

The Submitting Author accepts and understands that any supply made under these terms is made by BMJ to the Submitting Author unless you are acting as an employee on behalf of your employer or a postgraduate student of an affiliated institution which is paying any applicable article publishing charge ("APC") for Open Access articles. Where the Submitting Author wishes to make the Work available on an Open Access basis (and intends to pay the relevant APC), the terms of reuse of such Open Access shall be governed by a Creative Commons licence – details of these licences and which Creative Commons licence will apply to this Work are set out in our licence referred to above.

Other than as permitted in any relevant BMJ Author's Self Archiving Policies, I confirm this Work has not been accepted for publication elsewhere, is not being considered for publication elsewhere and does not duplicate material already published. I confirm all authors consent to publication of this Work and authorise the granting of this licence.

For peer review only

Protocol for a scoping review to understand what is known about how GPs make decisions with, for and on behalf of patients who lack capacity.

Simon Jack Ogden (Correspondence author)

jack.ogden@bristol.ac.uk

Centre for Ethics in Medicine, Population Health Sciences, Bristol Medical School, Canynge Hall, 39 Whatley Road, Bristol, BS8 1PS, UK

Richard Huxtable

r.huxtable@bristol.ac.uk

Centre for Ethics in Medicine, Population Health Sciences, Bristol Medical School, Canynge Hall, 39 Whatley Road, Bristol, BS8 1PS, UK

Jonathan Ives

j.ives@bristol.ac.uk

Centre for Ethics in Medicine, Population Health Sciences, Bristol Medical School, Canynge Hall, 39 Whatley Road, Bristol, BS8 1PS, UK

Keywords: Best interests, Incapacity, Decision-making, Empirical bioethics

For peer review only

ABSTRACT

Introduction

GPs and allied health care professionals working in primary care are regularly required to make decisions with, for and on behalf of patients who lack capacity. In England and Wales these decisions are made for incapacitated adult patients under the Mental Capacity Act 2005 (MCA), which primarily requires that decisions are made in the patient's "best interests". Regarding children, decisions are also made in their best interests but are done so under the Children Act 1989, which places paramount importance on the welfare of the child. Decisions for children are usually made by parents, but a GP may become involved if they feel a parent is not acting in the best interests of the child. Internationally, including elsewhere in the UK, different approaches are taken. We hypothesise that, despite the legislation and professional guidelines, there are many different approaches taken by GPs and allied healthc are professionals in England and Wales when making these complex decisions with, for and on behalf of patients who lack capacity. To better understand what is known about how these decisions are made, we plan to undertake a scoping review and directed content analysis of the literature. While the majority of decisions made in primary care are made by GPs, for completeness, this review will include all allied health care professionals working in primary care.

Methods and analysis

To ensure a wide breadth of literature is captured, a scoping review will be undertaken as described by Arksey and O'Malley (2005). A five-stage approach will be taken when conducting this review: (1) Identifying the research question; (2) Identifying relevant papers; (3) Study selection; (4) Data extraction; and (5) Summarising and Synthesis. The final stage will include a directed content analysis of the data to help establish the cross-cutting themes.

Ethics and dissemination

The scoping review will be disseminated through conferences and peer-reviewed publications. This scoping review is the first (mapping) phase in a proposed larger study to explore how GPs make decisions with, for and on behalf of those who lack capacity. Qualitative research with GPs, patients and their families will follow, before all the results are synthesised using an "empirical bioethics" methodology.

STRENGTHS AND LIMITATIONS OF THIS STUDY

- This scoping review will map the current understanding of how decisions are or should be made in primary care with, for and on behalf of patients who lack capacity
- Arksey and O'Malley's scoping review methodology will be used and it will follow the PRISMA Extension for Scoping Reviews (PRISMA-ScR) checklist.
- An international search will allow comparison of approaches between different legal jurisdictions.
- The data will be critically synthesised with reference to ethical and legal principles to identify cross-cutting themes and to ascertain where further research is needed on this increasingly important topic.

INTRODUCTION

GPs in the UK National Health Service provide over 300 million consultations per year[1] and the workload continues to rise year-on-year.[2,3] It has been said that the NHS is a victim of its own success; with novel and improved treatments, patients live longer, albeit with higher rates of co-

For peer review only

morbidity and medical complexity, often with reduced capacity to make their own medical decisions.[4] As a result, GPs are seeing an increasing number of patients who are unable to make their own decisions about their medical treatment.[5] Some common conditions that often result in decisions being made on behalf of the patient include dementia, learning disabilities, acquired traumatic brain injuries and mental health conditions. In addition to medical decisions, GPs are also often involved in decisions concerning the location of a patient's care, be that at home, in hospital or in a care home.

In England and Wales, the Mental Capacity Act 2005 (MCA)[6] provides a legal framework for making decisions for patients who lack capacity i.e. are unable to do so themselves. The assessment of capacity under the MCA can be made by any professional involved in the patient's care; in primary care, this will often be a GP, although in care homes, for example, this may be an allied healthcare professional. The MCA sets out a two-stage test of capacity. Firstly, the diagnostic test asks whether the person has an impairment of their mind or brain, whether as a result of an illness, or external factors such as alcohol or drug use. Secondly, the functional test asks whether the impairment means that the person is unable to make a specific decision when they need to.

The MCA primarily states that if a patient lacks the capacity to make a particular decision, then it can be made for them in their "best interests" and must be done in a way that is least restrictive of their rights. Although the Act does not provide a formula for best interests decision-making, the Act does disclose some of the factors that must be considered when making these best interest decisions, including the patient's past and present wishes and feelings, the beliefs and values that would likely influence their decision if they had capacity and other factors that they would be likely to consider if they were able to do so. There is also a duty to consult with those close to the patient, such as their family, when making best interests decisions.

The MCA also empowers patients to make provision for a future in which they may lack the capacity to make decisions. Two options are available. First, the MCA provides for proxy decision-making by introducing "lasting power of attorneys", who can make decisions on behalf of patients when they lose capacity. However, these proxies must ultimately still conform to the rules set out in the MCA with regards to acting in the patient's best interests. Second, the MCA places advance decision-making on a statutory footing, by introducing the "advance decision to refuse treatment", which may refuse (but not consent to or demand) specified treatments that might otherwise be provided after the patient has lost capacity.

Anglo-Welsh law regarding the capacity to consent and refuse treatment in children is slightly more complicated. The treatment of children is generally governed by the Children Act 1989 (CA), which requires that the paramount consideration is the welfare, or "best interests", of the child. The Family Law Reform Act 1969 states that those aged 16 and 17 years of age have the same legal ability to consent as an adult, however this does not mean they have a right to refuse treatment (Re W (A Minor) [7] and indeed someone with parental responsibility (or a court) may consent on their behalf. In children younger than 16, the law surrounding their capacity to consent is set out in Gillick v West Norfolk and Wisbech AHA.[8] The term "Gillick competence" refers to a child under 16 who "reaches a sufficient understanding and intelligence to be capable of making up his own mind on the matter requiring decision".[9] As with older children, even a Gillick competent minor cannot expect his refusal of treatment to operate as a veto. As such, where a child refuses to consent, or lacks the competence to consent, decisions may be made by others, in the "best interests" of the child.

Internationally, different legal jurisdictions provide different approaches to making decisions with, for and on behalf of patients who lack capacity to make these decisions themselves. For example, in

For peer review only

Scotland, patients are treated under the Adults with Incapacity (Scotland) Act 2000 where a doctor is required to complete a 'section 47' certificate if a patient is deemed to lack capacity and cannot use force unless immediately necessary. Conspicuous is the absence of the term "best interests" in the Adults with Incapacity (Scotland) Act, instead it chooses to set out five principles which must be applied when making decisions. Any action or decision must benefit the person, it must be the least-restrictive option, take account of the wishes of the person, include a consultation with a relevant other and encourage the person to use existing skills and develop new skills. In Northern Ireland, patients are treated under the Mental Capacity (Northern Ireland) Act 2016 which covers the treatment of all patients who lack capacity, including those in whom incapacity arises as a result of a mental health condition. This is different to England and Wales, where there is separate legislation – the Mental Health Act 2007 – governing the treatment of those with mental disorders. It is, however, similar in that it uses the 'best interests' test to guide how these decisions should be made. In the US, decisions may be made by a nominated surrogate decision-maker, such as a family member.[10]

The United Nations Convention on the Rights of Persons with Disabilities (CRPD) is also of importance given that it was ratified by the United Kingdom in 2009 and is hailed by some as advocating for a supported decision making approach [11] rather than a substituted or surrogate decision making approach of which best interests decisions are an example. The UN Committee on the Rights of Persons with Disabilities stated that 'perceived or actual deficits in mental capacity must not be used as justification for denying legal capacity.'[12] It is explicitly stated by this UN committee that best interests decisions are substituted decisions and should be replaced by supported decisions.

Returning to England and Wales, the law on best interest decisions has been further criticised as being vague, making its application open to divergent interpretations.[13] Neither Act – the MCA or the CA – *elaborates* a particular account of what is in a patient's best interests; rather, a non-exhaustive list of different factors is *enumerated*.[14,15] Different stakeholders and proxy decision-makers – such as clinicians, those with lasting powers of attorney, or parents – might point to different factors and reach different decisions, even in seemingly similar cases. We hypothesise that, despite the legislation and professional guidelines,[16,17,18] there are many different approaches taken by GPs and allied healthcare professionals when making these complex decisions depending on their interpretation of the facts, their own moral judgement, and their own understanding of the patient in front of them, with some (but not all) of whom they might have a longstanding relationship.

STUDY RATIONALE

With the increasing incidence of patients [5] who lack capacity to make decisions themselves comes an increasing need to understand how these decisions are made by GPs and allied healthcare professionals and how this process might be improved.

GPs have a unique role in the healthcare of patients and are often involved in the care of both the patient and their family, which can sometimes be for many years, from childhood to adulthood. GPs are also responsible for coordinating a patient's secondary care and managing most of their chronic diseases in the community setting.[19] While continuity of care has suffered in recent years, there is still a continuity in the relationship between GP and patient, which makes GPs well-placed to be involved in many of the complex medical decisions for patients who lack capacity.[20] The decisions being made could range from what treatment the patient should receive at the end of their life to whether a patient should be admitted to hospital or a care home.[21] Given these are often

For peer review only

important decisions that have a significant impact both for the patient and their family, it is important to understand how and why these decisions are made. We hope to find out what is understood about how decisions are or should be made in primary care with, for and on behalf of patients who lack capacity.

STUDY OBJECTIVES

The objective of this scoping review is to identify and map current research on how decisions are made with, for and on behalf of patients who lack capacity in primary care. The main concepts, approaches and theories will be established and synthesised with reference to current ethical principles and the various legal positions internationally. The overall objective is to identify knowledge gaps in the literature and areas where further research is needed.

METHODS AND ANALYSIS

Protocol design

A scoping review will be performed as described by Arksey and O'Malley [22] and set out in the PRISMA guidelines by Tricco et al.[23] A scoping review methodology has been chosen as it will allow us to identify what is known about the key characteristics and factors that are important to decisions made with, for and on behalf of patients who lack capacity. Following initial searches, it was clear that there is limited research currently published in this area, making a scoping review an appropriate approach. Scoping reviews map the topic as a whole and highlight where gaps in knowledge exist, thus helping guide further research.[24] The aim is to understand the breadth and depth of existing research knowledge.[25]

According to Arksey and O'Malley, there are six stages to undertaking a scoping review: identify the research question; identify relevant studies; study selection; data collection; summarising; and finally, expert consultation. The first five of these stages will be applied in this scoping review. The final consultation stage will be omitted as this will form part of the next phase of the research.

Stage 1: Identify research question

The research question was formulated in consultation with the research team and with consideration given to the further planned "empirical bioethics" research. "Empirical bioethics" combines theoretical and empirical research and may take various forms, but here will involve mapping what is currently known through literature reviews (this phase),[26]empirical research to deepen our understanding, then shaping work, to recommend ways forward.[27] The question posed for the current mapping phase was intentionally kept broad in its wording so as to capture all the published research that speaks to this question, including both empirical and theoretical research. The main research question is:

- What is understood about how decisions are or should be made in primary care with, for and on behalf of patients who lack capacity?

Subordinate research questions include:

1. What guidelines, approaches and processes are used in making decisions in primary care with, for and on behalf of patients who lack capacity?
2. Who participates in, or is consulted about, decisions in primary care with, for and on behalf of patients who lack capacity?
3. Which factors are considered by the participants when making decisions in primary care with, for and on behalf of patients who lack capacity?

For peer review only

4. What does the theoretical literature reveal about how decisions are or should be made in primary care with, for and on behalf of patients who lack capacity?

Stage 2: Identify relevant studies – search strategy

A limited initial search was carried out using MEDLINE (PubMed) and Web of Science to discover frequently used search terms, abbreviations, keywords and MeSH terms. This stage is important so that terms that might be used in different countries and disciplines are discovered and included in the final search string to capture all relevant research. A subject librarian also advised on the proposed search terms and approach.

Pilot searches were performed to test various search strings using different combinations of keywords in addition to wildcards (*) and Boolean modifiers to produce the final search string. The aim of the pilot searches was to capture as much of the relevant research as possible.

The article title and abstracts of papers will be searched in MEDLINE, Embase, PsychINFO, The Cochrane Library, Web of Science and JSTOR using the terms in the final search string:

[gp OR general pract* OR primary care] AND
[capacity or compet* or incapacit*] AND
[best interest* or decision making or decision]

Stage 3: Study selection

The title and abstracts of all the papers returned from the search will be transferred to Endnote software where they will be screened by the lead reviewer (SJO) according to the inclusion and exclusion criteria below. Papers will be classified into "include", "exclude" and "unsure" groups. Duplicated papers will be removed.

A second reviewer (RH) will screen 10% of the included and excluded papers to aid reliability. The second reviewer will also review all those papers in the "unsure" group. Any disagreements that cannot be resolved by discussion will be resolved by bringing in a third reviewer (JI). The team will meet regularly to discuss results and will seek to reach consensus on the papers to include and exclude. Where consensus is not reached, a majority decision will be carried (i.e. include or exclude).

Once the list of included papers is agreed, the first reviewer will screen the references of included papers to check for further papers that might fit the inclusion criteria. Any additional papers found will repeat the process above, as needed.

All papers that are found to fit the inclusion and exclusion criteria will be included in the scoping review. Given that this is a time-limited scoping review looking at the breadth of research rather than the quality of the research, all papers found to fit the inclusion criteria will be included in the review.

Inclusion

- Peer-reviewed papers, books, book chapters, national guidelines.
- All types of empirical research including qualitative, quantitative and mixed methods.
- Theoretical studies.
- Any paper that addresses the question of *how* medical decisions are made for patients who lack capacity and *what* factors are considered important. It is not necessary for this to be the paper's primary aim.
- Papers situated in the primary healthcare setting.

For peer review only

- Papers that consider decisions about healthcare treatment/non-treatment including decision about the location of a patients care.
- Papers where decisions are made following advance care planning when patients were considered to have capacity.
- Any papers published since 1960 and in English.

Exclusion

- Grey literature including journalism.
- Papers that do not refer to lack of capacity or, in the case of a child, competence when referring to any medical decision making.
- Papers not situated in the primary healthcare setting.
- Papers that do not consider decisions about healthcare treatment/non-treatment e.g. that concern research and/or social care exclusively.Papers not in English.

Stage 4: Data extraction and analysis

A directed content analysis will be undertaken in order to identify and describe key themes present in the papers. A directed content analysis begins with a pre-established coding framework, but then allows new codes to be used, or existing codes to be further specified, as the analysis progresses.[28] Directed content analysis will be performed so that the analysis can be directly guided by the search for ethical material – with codes relating to ethical content around best interests decisions. These codes will be developed by the first reviewer with regular discussion with the second and third reviewers. Where there are disagreements over coding the majority decision will be carried. While synthesis of the data is not always performed in scoping reviews, it was felt it would add context to the research included in the review.

The first stage will be data extraction (or 'charting the results') - an iterative process that involves extracting predefined data into a charting sheet, which can then be used to explore themes. The draft charting table comprises key details about the papers, but also includes the initial coding framework that will be applied to papers - focussing on 4 areas of results (see vii below). A description of what those papers say about each of those areas will be entered into the data extraction sheet, and then further sub codes will be developed, as appropriate, to further distinguish between, and record, different kinds of content.

i. Articles details
ii. Country of origin
iii. Broad category (i.e. patient situation/condition)
iv. Brief summary
v. Aims of study
vi. Methods/Type of Study
vii. Results
    a. Approaches and processes used in making decisions
    b. Participants involved in making decisions
    c. Factors considered in making decisions
    d. Theoretical argument(s) about how to make decisions
viii. Conclusion
ix. Reflections from reviewer

The first reviewer (SJO) will extract data from all included empirical and theoretical research into the charting table. Data from quantitative research will be described and charted. To improve reliability,

For peer review only

10-15% of papers will also have data extracted independently by a second (RH) and third (JI) reviewer.

The extracted data and expanded coding will be discussed amongst the group early in the data extraction stage to discuss disagreements and agree on how the coding framework should be developed and applied.

Stage 5: Summarising and synthesis

The second phase in the analysis will be to systematically explore the codes developed (and data extracted) in stage 4 to develop larger descriptive themes which summarise what the evidence from the papers show.

Any quantitative evidence will be simply described and reported narratively.

Finally, consideration will be given to what gaps in the literature exist and how these might inform further research on the topic.

PATIENT AND PUBLIC INVOLVEMENT

No patients involved.

ETHICS AND DISSEMINATION

One paper conveying the results of the review will be prepared for publication in a peer reviewed journal. The authors will also seek to present the findings at appropriate bioethics or primary care conferences in the UK. The results from this review will help guide the next phase of the research involving a qualitative "empirical bioethics" study, looking further into how GPs do and should make these decisions.

No ethics approval is required for this scoping review.

REFERENCES

1. NSH Five Year Forward View. 2014 https://www.england.nhs.uk/five-year-forward-view/next-steps-on-the-nhs-five-year-forward-view/primary-care/. Accessed December 2019
2. Hobbs FDR, et al. Clinical workload in UK primary care: a retrospective analysis of 100 million consultations in England. *Lancet* 2016 Jun 4;387(10035):2323-2330. doi:10.1016/S0140-6736(16)00620-6
3. Lay-Flurrie S, Mathieu E, Bankhead C, et al. Patient consultation rate and clinical and NHS outcomes: a cross-sectional analysis of English primary care data from 2.7 million patients in 238 practices. *BMC Health Serv Res* 2019;19(1):219. doi:10.1186/s12913-019-4036-y
4. Wallace E, Salisbury C, Guthrie B, et al. Managing patients with multimorbidity in primary care *BMJ* 2015; 350 :h176. doi:10.1136/bmj.h176
5. Wancata J, Musalek M, Alexandrowicz R, et al. Number of dementia sufferers in Europe between the years 2000 and 2050. *Eur Psychiatry* 2003;18:306–13.
6. The Mental Capacity Act 2005
7. Re W (Minors) [2016] EWHC 2226 (Fam).
8. Gillick v West Norfolk & Wisbeck Area Health Authority [1986] AC 112 House of Lords.
9. [1986] AC 112 at page 189.
10. Torke AM, Alexander GC, & Lantos J. Substituted Judgment: The Limitations of Autonomy in Surrogate Decision Making. *J Gen Intern Med* 2008; 23:1514–1517. doi:10.1007/s11606-008-0688-8

11. Kohn N, Blumenthal JA, Campbell AT. Supported Decision-Making: A Viable Alternative to Guardianship?. *Penn State Law Review* 2013;117:4 1111-1157 at 1129
12. UN Committee on the Rights of Persons with Disabilities. General comment no. 1, article 12: Equal recognition before the law, CRPD/C/GC/1 http://daccessdds-ny.un.org/doc/UNDOC/GEN/G14/031/20/PDF/G1403120.pdf
13. DeMartino ES, Dudzinski DM, Doyle CK, et al. Who Decides When a Patient Can't? Statutes on Alternate Decision Makers. *N Engl J Med* 2017;376(15):1478–1482. doi:10.1056/NEJMms1611497
14. Martin W, Freyenhagen F, Hall E, et al. An unblinkered view of best interests. *BMJ* 2012;345 :e8007. doi: 10.1136/bmj.e8007
15. Lim CM, Dunn MC, Chin JJ. Clarifying the best interests standard: the elaborative and enumerative strategies in public policy-making. *J Med Ethics* 2016;42:542–54. doi:10.1136/medethics-2016-103454
16. GMC Good medical practice (2013). General Medical Council http://www.gmc-uk.org/guidance/good_medical_practice.asp.
17. Best interests decision-making for adults who lack capacity A toolkit for doctors working in England and Wales. BMA.
18. General Medical Council: 0–18 years: guidance for all doctors. www.gmc-uk.org/ethicalguidance
19. Bodenheimer T, Wagner EH, Grumbach K. Improving primary care for patients with chronic illness: the chronic care model, part 2. *JAMA* 2002; 288: 1909–14. doi:10.1001/jama.288.15.1909
20. Campbell S M, Hann M, Hacker J, et al. Identifying predictors of high quality care in English general practice: observational study. *BMJ* 2001;323:784. doi:10.1136/bmj.323.7316.784
21. Tammes, P, Payne, RA, Salisbury, C, et al. The impact of a named GP scheme on continuity of care and emergency hospital admission: A cohort study among older patients in England, 2012-2016. *BMJ Open* 2019;vol.9,no.9,e029103. doi:10.1136/bmjopen-2019-029103
22. Arksey H, O'Malley L. Scoping studies: Towards a Methodological Framework. *Int J Soc Res* 2005;8:1,19-32. doi:10.1080/1364557032000119616
23. Tricco AC, et al. PRISMA Extension for Scoping Reviews (PRISMA-ScR): Checklist and Explanation. *Ann Intern Med* 2018 Oct 2;169(7):467-473. doi:10.7326/M18-0850
24. Peters, MDJ, Godfrey, C, Kahlil, H, et al. Guidance for conducting systematic scoping reviews. *Int J Evid Based Healthc* 2015;vol.13,ed.3,pp.141-46. doi:10.1097/XEB.0000000000000050
25. Levac D, Colquhoun H, O'Brien KK. Scoping studies: advancing the methodology. *Implement Sci* 2010;5(1):1. doi: 10.1186/1748-5908-5-6
26. Huxtable, R., Ives, J. Mapping, framing, shaping: a framework for empirical bioethics research projects. *BMC Med Ethics* 2019;20,86. doi:10.1186/s12910-019-0428-0
27. Ives, Dunn, Cribb. Empirical Bioethics: Theoretical and Practical Perspectives. Cambridge University Press 2016.
28. Hsieh HS, & Shannon S. Three Approaches to Qualitative Content Analysis. *Qualitative health research* 2005;15. 1277-88. doi:10.1177/1049732305276687

AUTHORS' CONTRIBUTIONS

SJO conceived the idea for the scoping review. SJO, JI and RH all contributed to development of the research questions and study design. SJO drafted the initial protocol with input from JI and RH. All authors contributed to re-drafting and subsequent final revision of this protocol. SJO is the guarantor of the review.

For peer review only

ACKNOWLEDGEMENTS

I would like to express my appreciation to Dr Giles Birchley, Dr Zuzana Deans and Dr Emanuele Valenti and Dr Iona Sherwood for the general advice, help and support they have given in conceiving this project.

FUNDING STATEMENT

This work was supported by the Elizabeth Blackwell Institute and Wellcome Trust Institutional Strategic Support Fund, grant number 204813/Z/16/Z. The Elizabeth Blackwell Institute and Wellcome Trust were not involved in the development of the protocol other than providing financial support.

COMPETING INTEREST STATEMENT

We have read and understood the BMJ policy on declaration of interests and declare that we have no competing interests.

WORD COUNT: 3051

For peer review only

Reporting checklist for protocol of a systematic review.

Based on the PRISMA-P guidelines.

Instructions to authors

Complete this checklist by entering the page numbers from your manuscript where readers will find each of the items listed below.

Your article may not currently address all the items on the checklist. Please modify your text to include the missing information. If you are certain that an item does not apply, please write "n/a" and provide a short explanation.

Upload your completed checklist as an extra file when you submit to a journal.

In your methods section, say that you used the PRISMA-Preporting guidelines, and cite them as:

Moher D, Shamseer L, Clarke M, Ghersi D, Liberati A, Petticrew M, Shekelle P, Stewart LA. Preferred Reporting Items for Systematic Review and Meta-Analysis Protocols (PRISMA-P) 2015 statement. Syst Rev. 2015;4(1):1.

		Reporting Item	Page Number
**Title**			
Identification	#1a	Identify the report as a protocol of a systematic review	1
Update	#1b	If the protocol is for an update of a previous systematic review, identify as such	n/a
**Registration**			
	#2	If registered, provide the name of the registry (such as PROSPERO) and registration number	n/a
**Authors**			
Contact	#3a	Provide name, institutional affiliation, e-mail address of all protocol authors; provide physical mailing address of corresponding author	1
Contribution	#3b	Describe contributions of protocol authors and identify the guarantor of the review	9
**Amendments**			

For peer review only

	#4	If the protocol represents an amendment of a previously completed or published protocol, identify as such and list changes; otherwise, state plan for documenting important protocol amendments	n/a
**Support**			
Sources	#5a	Indicate sources of financial or other support for the review	9
Sponsor	#5b	Provide name for the review funder and / or sponsor	9
Role of sponsor or funder	#5c	Describe roles of funder(s), sponsor(s), and / or institution(s), if any, in developing the protocol	n/a
**Introduction**			
Rationale	#6	Describe the rationale for the review in the context of what is already known	4
Objectives	#7	Provide an explicit statement of the question(s) the review will address with reference to participants, interventions, comparators, and outcomes (PICO)	4
**Methods**			
Eligibility criteria	#8	Specify the study characteristics (such as PICO, study design, setting, time frame) and report characteristics (such as years considered, language, publication status) to be used as criteria for eligibility for the review	5
Information sources	#9	Describe all intended information sources (such as electronic databases, contact with study authors, trial registers or other grey literature sources) with planned dates of coverage	6
Search strategy	#10	Present draft of search strategy to be used for at least one electronic database, including planned limits, such that it could be repeated	5
Study records - data management	#11a	Describe the mechanism(s) that will be used to manage records and data throughout the review	7
Study records - selection process	#11b	State the process that will be used for selecting studies (such as two independent reviewers) through each phase of the review (that is, screening, eligibility and inclusion in meta-analysis)	6
Study records - data collection process	#11c	Describe planned method of extracting data from reports (such as piloting forms, done independently, in duplicate), any processes for	7

For peer review only

		obtaining and confirming data from investigators	
Data items	#12	List and define all variables for which data will be sought (such as PICO items, funding sources), any pre-planned data assumptions and simplifications	6
Outcomes and prioritization	#13	List and define all outcomes for which data will be sought, including prioritization of main and additional outcomes, with rationale	7
Risk of bias in individual studies	#14	Describe anticipated methods for assessing risk of bias of individual studies, including whether this will be done at the outcome or study level, or both; state how this information will be used in data synthesis	7
Data synthesis	#15a	Describe criteria under which study data will be quantitatively synthesised	7
Data synthesis	#15b	If data are appropriate for quantitative synthesis, describe planned summary measures, methods of handling data and methods of combining data from studies, including any planned exploration of consistency (such as I2, Kendall's τ)	7
Data synthesis	#15c	Describe any proposed additional analyses (such as sensitivity or subgroup analyses, meta-regression)	7
Data synthesis	#15d	If quantitative synthesis is not appropriate, describe the type of summary planned	n/a
Meta-bias(es)	#16	Specify any planned assessment of meta-bias(es) (such as publication bias across studies, selective reporting within studies)	n/a
Confidence in cumulative evidence	#17	Describe how the strength of the body of evidence will be assessed (such as GRADE)	n/a

The PRISMA-P checklist is distributed under the terms of the Creative Commons Attribution License CC-BY 4.0. This checklist was completed on 25. February 2020 using https://www.goodreports.org/, a tool made by the EQUATOR Network in collaboration with Penelope.ai

For peer review only

Protocol for a scoping review to understand what is known about how GPs make decisions with, for and on behalf of patients who lack capacity

Journal:	*BMJ Open*
Manuscript ID	bmjopen-2020-038032.R2
Article Type:	Protocol
Date Submitted by the Author:	05-Sep-2020
Complete List of Authors:	Ogden, Simon; University of Bristol, Centre for Ethics in Medicine<br>Huxtable, Richard; University of Bristol, School of Social and Community Medicine<br>Ives, Jonathan; University of Bristol, Centre for Ethics in Medicine
**Primary Subject Heading**:	General practice / Family practice
Secondary Subject Heading:	Ethics, Mental health, Paediatrics, Patient-centred medicine, Research methods
Keywords:	PRIMARY CARE, MEDICAL ETHICS, QUALITATIVE RESEARCH, STATISTICS & RESEARCH METHODS

I, the Submitting Author has the right to grant and does grant on behalf of all authors of the Work (as defined in the below author licence), an exclusive licence and/or a non-exclusive licence for contributions from authors who are: i) UK Crown employees; ii) where BMJ has agreed a CC-BY licence shall apply, and/or iii) in accordance with the terms applicable for US Federal Government officers or employees acting as part of their official duties; on a worldwide, perpetual, irrevocable, royalty-free basis to BMJ Publishing Group Ltd ("BMJ") its licensees and where the relevant Journal is co-owned by BMJ to the co-owners of the Journal, to publish the Work in this journal and any other BMJ products and to exploit all rights, as set out in our licence.

The Submitting Author accepts and understands that any supply made under these terms is made by BMJ to the Submitting Author unless you are acting as an employee on behalf of your employer or a postgraduate student of an affiliated institution which is paying any applicable article publishing charge ("APC") for Open Access articles. Where the Submitting Author wishes to make the Work available on an Open Access basis (and intends to pay the relevant APC), the terms of reuse of such Open Access shall be governed by a Creative Commons licence – details of these licences and which Creative Commons licence will apply to this Work are set out in our licence referred to above.

Other than as permitted in any relevant BMJ Author's Self Archiving Policies, I confirm this Work has not been accepted for publication elsewhere, is not being considered for publication elsewhere and does not duplicate material already published. I confirm all authors consent to publication of this Work and authorise the granting of this licence.

For peer review only

Protocol for a scoping review to understand what is known about how GPs make decisions with, for and on behalf of patients who lack capacity.

Simon Jack Ogden (Correspondence author)

jack.ogden@bristol.ac.uk

Centre for Ethics in Medicine, Population Health Sciences, Bristol Medical School, Canynge Hall, 39 Whatley Road, Bristol, BS8 1PS, UK

Richard Huxtable

r.huxtable@bristol.ac.uk

Centre for Ethics in Medicine, Population Health Sciences, Bristol Medical School, Canynge Hall, 39 Whatley Road, Bristol, BS8 1PS, UK

Jonathan Ives

j.ives@bristol.ac.uk

Centre for Ethics in Medicine, Population Health Sciences, Bristol Medical School, Canynge Hall, 39 Whatley Road, Bristol, BS8 1PS, UK

Keywords: Best interests, Incapacity, Decision-making, Empirical bioethics

For peer review only

ABSTRACT

Introduction

GPs and allied health care professionals working in primary care are regularly required to make decisions with, for and on behalf of patients who lack capacity. In England and Wales these decisions are made for incapacitated adult patients under the Mental Capacity Act 2005 (MCA), which primarily requires that decisions are made in the patient's "best interests". Regarding children, decisions are also made in their best interests but are done so under the Children Act 1989, which places paramount importance on the welfare of the child. Decisions for children are usually made by parents, but a GP may become involved if he or she feels a parent is not acting in the best interests of the child. Internationally, including elsewhere in the UK, different approaches are taken. We hypothesise that, despite the legislation and professional guidelines, there are many different approaches taken by GPs and allied healthcare professionals in England and Wales when making these complex decisions with, for and on behalf of patients who lack capacity. To better understand what is known about how these decisions are made, we plan to undertake a scoping review and directed content analysis of the literature. While the majority of decisions made in primary care are made by GPs, for completeness, this review will include all allied health care professionals working in primary care.

Methods and analysis

To ensure a wide breadth of literature is captured, a scoping review will be undertaken as described by Arksey and O'Malley (2005). A five-stage approach will be taken when conducting this review: (1) Identifying the research question; (2) Identifying relevant papers; (3) Study selection; (4) Data extraction; and (5) Summarising and Synthesis. The final stage will include a directed content analysis of the data to help establish the cross-cutting themes.

Ethics and dissemination

The scoping review will be disseminated through conferences and peer-reviewed publications. This scoping review is the first (mapping) phase in a proposed larger study to explore how GPs make decisions with, for and on behalf of those who lack capacity. Qualitative research with GPs, patients and their families will follow, before all the results are synthesised using an "empirical bioethics" methodology.

STRENGTHS AND LIMITATIONS OF THIS STUDY

- This scoping review will map the current understanding of how decisions are or should be made in primary care with, for and on behalf of patients who lack capacity
- Arksey and O'Malley's scoping review methodology will be used and it will follow the PRISMA Extension for Scoping Reviews (PRISMA-ScR) checklist.
- An international search will allow comparison of approaches between different legal jurisdictions.
- The data will be critically synthesised with reference to ethical and legal principles to identify cross-cutting themes and to ascertain where further research is needed on this increasingly important topic.

INTRODUCTION

GPs in the UK National Health Service provide over 300 million consultations per year[1] and the workload continues to rise year-on-year.[2,3] It has been said that the NHS is a victim of its own

For peer review only

success; with novel and improved treatments, patients live longer, albeit with higher rates of co-morbidity and medical complexity, often with reduced capacity to make their own medical decisions.[4] As a result, GPs are seeing an increasing number of patients who are unable to make their own decisions about their medical treatment.[5] Some common conditions that often result in decisions being made on behalf of the patient include dementia, learning disabilities, acquired traumatic brain injuries and mental health conditions. In addition to medical decisions, GPs are also often involved in decisions concerning the location of a patient's care, be that at home, in hospital or in a care home.

In England and Wales, the Mental Capacity Act 2005 (MCA)[6] provides a legal framework for making decisions for patients who lack capacity i.e. are unable to do so themselves. The assessment of capacity under the MCA can be made by any professional involved in the patient's care; in primary care, this will often be a GP, although in care homes, for example, this may be an allied healthcare professional. The MCA sets out a two-stage test of capacity. Firstly, the diagnostic test asks whether the person has an impairment of their mind or brain, whether as a result of an illness, or external factors such as alcohol or drug use. Secondly, the functional test asks whether the impairment means that the person is unable to make a specific decision when they need to.

The MCA primarily states that if a patient lacks the capacity to make a particular decision, then it can be made for him or her in his or her "best interests" and must be done in a way that is least restrictive of their rights. Although the Act does not provide a formula for best interests decision-making, the Act does disclose some of the factors that must be considered when making these best interest decisions, including the patient's past and present wishes and feelings, the beliefs and values that would likely influence their decision if they had capacity and other factors that they would be likely to consider if they were able to do so. There is also a duty to consult with those close to the patient, such as his or her family, when making best interests decisions.

The MCA also empowers patients to make provision for a future in which they may lack the capacity to make decisions. Two options are available. First, the MCA provides for proxy decision-making by introducing "lasting power of attorneys", who can make decisions on behalf of patients when they lose capacity. However, these proxies must ultimately still conform to the rules set out in the MCA with regards to acting in the patient's best interests. Second, the MCA places advance decision-making on a statutory footing, by introducing the "advance decision to refuse treatment", which may refuse (but not consent to or demand) specified treatments that might otherwise be provided after the patient has lost capacity.

Anglo-Welsh law regarding the capacity to consent and refuse treatment in children is slightly more complicated. The treatment of children is generally governed by the Children Act 1989 (CA), which requires that the paramount consideration is the welfare, or "best interests", of the child. The Family Law Reform Act 1969 states that those aged 16 and 17 years of age have the same legal ability to consent as an adult, however this does not mean they have a right to refuse treatment (Re W (A Minor) [7] and indeed someone with parental responsibility (or a court) may consent on their behalf. In children younger than 16, the law surrounding their capacity to consent is set out in Gillick v West Norfolk and Wisbech AHA.[8] The term "Gillick competence" refers to a child under 16 who "reaches a sufficient understanding and intelligence to be capable of making up his own mind on the matter requiring decision".[9] As with older children, even a Gillick competent minor cannot expect his refusal of treatment to operate as a veto. As such, where a child refuses to consent, or lacks the competence to consent, decisions may be made by others, in the "best interests" of the child.

For peer review only

Internationally, different legal jurisdictions provide different approaches to making decisions with, for and on behalf of patients who lack capacity to make these decisions themselves. For example, in Scotland, patients are treated under the Adults with Incapacity (Scotland) Act 2000 where a doctor is required to complete a 'section 47' certificate if a patient is deemed to lack capacity and cannot use force unless immediately necessary. Conspicuous is the absence of the term "best interests" in the Adults with Incapacity (Scotland) Act, instead it chooses to set out five principles which must be applied when making decisions. Any action or decision must benefit the person, it must be the least-restrictive option, take account of the wishes of the person, include a consultation with a relevant other and encourage the person to use existing skills and develop new skills. In Northern Ireland, patients are treated under the Mental Capacity (Northern Ireland) Act 2016 which covers the treatment of all patients who lack capacity, including those in whom incapacity arises as a result of a mental health condition. This is different to England and Wales, where there is separate legislation – the Mental Health Act 2007 – governing the treatment of those with mental disorders. It is, however, similar in that it uses the 'best interests' test to guide how these decisions should be made. In the US, decisions may be made by a nominated surrogate decision-maker, such as a family member.[10]

The United Nations Convention on the Rights of Persons with Disabilities (CRPD) is also of importance given that it was ratified by the United Kingdom in 2009 and is hailed by some as advocating for a supported decision making approach [11] rather than a substituted or surrogate decision making approach of which best interests decisions are an example. The UN Committee on the Rights of Persons with Disabilities stated that 'perceived or actual deficits in mental capacity must not be used as justification for denying legal capacity.'[12] It is explicitly stated by this UN committee that best interests decisions are substituted decisions and should be replaced by supported decisions.

Returning to England and Wales, the law on best interest decisions has been further criticised as being vague, making its application open to divergent interpretations.[13] Neither Act – the MCA or the CA – *elaborates* a particular account of what is in a patient's best interests; rather, a non-exhaustive list of different factors is *enumerated*.[14,15] Different stakeholders and proxy decision-makers – such as clinicians, those with lasting powers of attorney, or parents – might point to different factors and reach different decisions, even in seemingly similar cases. We hypothesise that, despite the legislation and professional guidelines,[16,17,18] there are many different approaches taken by GPs and allied healthcare professionals when making these complex decisions depending on their interpretation of the facts, their own moral judgement, and their own understanding of the patient in front of them, with some (but not all) of whom they might have a longstanding relationship.

STUDY RATIONALE

With the increasing incidence of patients [5] who lack capacity to make decisions themselves comes an increasing need to understand how these decisions are made by GPs and allied healthcare professionals and how this process might be improved.

GPs have a unique role in the healthcare of patients and are often involved in the care of both the patient and their family, which can sometimes be for many years, from childhood to adulthood. GPs are also responsible for coordinating a patient's secondary care and managing most of their chronic diseases in the community setting.[19] While continuity of care has suffered in recent years, there is still a continuity in the relationship between GP and patient, which makes GPs well-placed to be involved in many of the complex medical decisions for patients who lack capacity.[20] The decisions

For peer review only

being made could range from what treatment the patient should receive at the end of their life to whether a patient should be admitted to hospital or a care home.[21] Given these are often important decisions that have a significant impact both for the patient and their family, it is important to understand how and why these decisions are made. The scoping review aims to find out what is understood about how decisions are or should be made in primary care with, for and on behalf of patients who lack capacity.

STUDY OBJECTIVES

The objective of this scoping review is to identify and map current research on how decisions are made with, for and on behalf of patients who lack capacity in primary care. The main concepts, approaches and theories will be established and synthesised with reference to current ethical principles and the various legal positions internationally. The overall objective is to identify knowledge gaps in the literature and areas where further research is needed.

METHODS AND ANALYSIS

Protocol design

A scoping review will be performed as described by Arksey and O'Malley [22] and set out in the PRISMA guidelines by Tricco et al.[23] A scoping review methodology has been chosen as it will allow us to identify what is known about the key characteristics and factors that are important to decisions made with, for and on behalf of patients who lack capacity. Following initial searches, it was clear that there is limited research currently published in this area, making a scoping review an appropriate approach. Scoping reviews map the topic as a whole and highlight where gaps in knowledge exist, thus helping guide further research.[24] The aim is to understand the breadth and depth of existing research knowledge.[25]

According to Arksey and O'Malley, there are six stages to undertaking a scoping review: identify the research question; identify relevant studies; study selection; data collection; summarising; and finally, expert consultation. The first five of these stages will be applied in this scoping review. The final consultation stage will be omitted as this will form part of the next phase of the research. The time frame for completing the scoping review is 6 months.

Stage 1: Identify research question

The research question was formulated in consultation with the research team and with consideration given to the further planned "empirical bioethics" research. "Empirical bioethics" combines theoretical and empirical research and may take various forms, but here will involve mapping what is currently known through literature reviews (this phase),[26]empirical research to deepen our understanding, then shaping work, to recommend ways forward.[27] The question posed for the current mapping phase was intentionally kept broad in its wording so as to capture all the published research that speaks to this question, including both empirical and theoretical research. The main research question is:

- What is understood about how decisions are or should be made in primary care with, for and on behalf of patients who lack capacity?

Subordinate research questions include:

1. What guidelines, approaches and processes are used in making decisions in primary care with, for and on behalf of patients who lack capacity?
2. Who participates in, or is consulted about, decisions in primary care with, for and on behalf of patients who lack capacity?

For peer review only - http://bmjopen.bmj.com/site/about/guidelines.xhtml

3. Which factors are considered by the participants when making decisions in primary care with, for and on behalf of patients who lack capacity?
4. What does the theoretical literature reveal about how decisions are or should be made in primary care with, for and on behalf of patients who lack capacity?

Stage 2: Identify relevant studies – search strategy

A limited initial search was carried out using MEDLINE (PubMed) and Web of Science to discover frequently used search terms, abbreviations, keywords and MeSH terms. This stage is important so that terms that might be used in different countries and disciplines are discovered and included in the final search string to capture all relevant research. A subject librarian also advised on the proposed search terms and approach.

Pilot searches were performed to test various search strings using different combinations of keywords in addition to wildcards (*) and Boolean modifiers to produce the final search string. The aim of the pilot searches was to capture as much of the relevant research as possible.

The article title and abstracts of papers will be searched in MEDLINE, Embase, PsychINFO, The Cochrane Library, Web of Science and JSTOR using the terms in the final search string:

[gp OR general pract* OR primary care] AND
[capacity or compet* or incapacit*] AND
[best interest* or decision making or decision]

Stage 3: Study selection

The title and abstracts of all the papers returned from the search will be transferred to Endnote software where they will be screened by the lead reviewer (SJO) according to the inclusion and exclusion criteria below. Papers will be classified into "include", "exclude" and "unsure" groups. Duplicated papers will be removed.

A second reviewer (RH) will screen 10% of the included and excluded papers to aid reliability. The second reviewer will also review all those papers in the "unsure" group. Any disagreements that cannot be resolved by discussion will be resolved by bringing in a third reviewer (JI). The team will meet regularly to discuss results and will seek to reach consensus on the papers to include and exclude. Where consensus is not reached, a majority decision will be carried (i.e. include or exclude).

Once the list of included papers is agreed, the first reviewer will screen the references of included papers to check for further papers that might fit the inclusion criteria. Any additional papers found will repeat the process above, as needed.

All papers that are found to fit the inclusion and exclusion criteria will be included in the scoping review.

Inclusion

- Peer-reviewed papers, books, book chapters, national guidelines.
- All types of empirical research including qualitative, quantitative and mixed methods.
- Theoretical studies.
- Commentary or opinion pieces in peer-reviewed journals.
- Any paper that addresses the question of *how* medical decisions are made for patients who lack capacity and *what* factors are considered important. It is not necessary for this to be the paper's primary aim.
- Papers situated in the primary healthcare setting.

For peer review only

- Papers that consider decisions about healthcare treatment/non-treatment including decision about the location of a patients care.
- Papers where decisions are made following advance care planning when patients were considered to have capacity.
- Any papers published since 1960 and in English.

Exclusion

- Grey literature including journalism.
- Commentary or opinion pieces not in peer-reviewed journals.
- Papers that do not refer to lack of capacity or, in the case of a child, competence when referring to any medical decision making.
- Papers not situated in the primary healthcare setting.
- Papers that do not consider decisions about healthcare treatment/non-treatment e.g. that concern research and/or social care exclusively.Papers not in English.

Stage 4: Data extraction and analysis

A directed content analysis will be undertaken in order to identify and describe key themes present in the papers. A directed content analysis begins with a pre-established coding framework, but then allows new codes to be used, or existing codes to be further specified, as the analysis progresses.[28] Directed content analysis will be performed so that the analysis can be directly guided by the search for ethical material – with codes relating to ethical content around best interests decisions. These codes will be developed by the first reviewer with regular discussion with the second and third reviewers. Where there are disagreements over coding the majority decision will be carried. While synthesis of the data is not always performed in scoping reviews, it was felt it would add context to the research included in the review.

The first stage will be data extraction (or 'charting the results') - an iterative process that involves extracting predefined data into a charting sheet, which can then be used to explore themes. The draft charting table comprises key details about the papers, but also includes the initial coding framework that will be applied to papers - focussing on 4 areas of results (see vii below). A description of what those papers say about each of those areas will be entered into the data extraction sheet, and then further sub codes will be developed, as appropriate, to further distinguish between, and record, different kinds of content.

i. Articles details
ii. Country of origin
iii. Broad category (i.e. patient situation/condition)
iv. Brief summary
v. Aims of study
vi. Methods/Type of Study
vii. Results
    a. Approaches and processes used in making decisions
    b. Participants involved in making decisions
    c. Factors considered in making decisions
    d. Theoretical argument(s) about how to make decisions
viii. Conclusion
ix. Reflections from reviewer

For peer review only

The first reviewer (SJO) will extract data from all included empirical and theoretical research into the charting table. Data from quantitative research will be described and charted. To improve reliability, 10-15% of papers will also have data extracted independently by a second (RH) and third (JI) reviewer.

The extracted data and expanded coding will be discussed amongst the group early in the data extraction stage to discuss disagreements and agree on how the coding framework should be developed and applied.

Stage 5: Summarising and synthesis

The second phase in the analysis will be to systematically explore the codes developed (and data extracted) in stage 4 to develop larger descriptive themes which summarise what the evidence from the papers show.

Any quantitative evidence will be simply described and reported narratively.

Finally, consideration will be given to what gaps in the literature exist and how these might inform further research on the topic.

PATIENT AND PUBLIC INVOLVEMENT

No patients involved.

ETHICS AND DISSEMINATION

One paper conveying the results of the review will be prepared for publication in a peer reviewed journal. The authors will also seek to present the findings at appropriate bioethics or primary care conferences in the UK or virtual conferences internationally. The results from this review will help guide the next phase of the research involving a qualitative “empirical bioethics” study, looking further into how GPs do and should make these decisions.

No ethics approval is required for this scoping review.

REFERENCES

1. NSH Five Year Forward View. 2014 https://www.england.nhs.uk/five-year-forward-view/next-steps-on-the-nhs-five-year-forward-view/primary-care/. Accessed December 2019
2. Hobbs FDR, et al. Clinical workload in UK primary care: a retrospective analysis of 100 million consultations in England. *Lancet* 2016 Jun 4;387(10035):2323-2330. doi:10.1016/S0140-6736(16)00620-6
3. Lay-Flurrie S, Mathieu E, Bankhead C, et al. Patient consultation rate and clinical and NHS outcomes: a cross-sectional analysis of English primary care data from 2.7 million patients in 238 practices. *BMC Health Serv Res* 2019;19(1):219. doi:10.1186/s12913-019-4036-y
4. Wallace E, Salisbury C, Guthrie B, et al. Managing patients with multimorbidity in primary care *BMJ* 2015; 350 :h176. doi:10.1136/bmj.h176
5. Wancata J, Musalek M, Alexandrowicz R, et al. Number of dementia sufferers in Europe between the years 2000 and 2050. *Eur Psychiatry* 2003;18:306–13.
6. The Mental Capacity Act 2005
7. Re W (Minors) [2016] EWHC 2226 (Fam).
8. Gillick v West Norfolk & Wisbeck Area Health Authority [1986] AC 112 House of Lords.
9. [1986] AC 112 at page 189.

For peer review only

10. Torke AM, Alexander GC, & Lantos J. Substituted Judgment: The Limitations of Autonomy in Surrogate Decision Making. *J Gen Intern Med* 2008; 23:1514–1517. doi:10.1007/s11606-008-0688-8
11. Kohn N, Blumenthal JA, Campbell AT. Supported Decision-Making: A Viable Alternative to Guardianship?. *Penn State Law Review* 2013;117:4 1111-1157 at 1129
12. UN Committee on the Rights of Persons with Disabilities. General comment no. 1, article 12: Equal recognition before the law, CRPD/C/GC/1 http://daccessdds-ny.un.org/doc/UNDOC/GEN/G14/031/20/PDF/G1403120.pdf
13. DeMartino ES, Dudzinski DM, Doyle CK, et al. Who Decides When a Patient Can't? Statutes on Alternate Decision Makers. *N Engl J Med* 2017;376(15):1478–1482. doi:10.1056/NEJMms1611497
14. Martin W, Freyenhagen F, Hall E, et al. An unblinkered view of best interests. *BMJ* 2012;345 :e8007. doi: 10.1136/bmj.e8007
15. Lim CM, Dunn MC, Chin JJ. Clarifying the best interests standard: the elaborative and enumerative strategies in public policy-making. *J Med Ethics* 2016;42:542–54. doi:10.1136/medethics-2016-103454
16. GMC Good medical practice (2013). General Medical Council http://www.gmc-uk.org/guidance/good_medical_practice.asp.
17. Best interests decision-making for adults who lack capacity A toolkit for doctors working in England and Wales. BMA.
18. General Medical Council: 0–18 years: guidance for all doctors. www.gmc-uk.org/ethicalguidance
19. Bodenheimer T, Wagner EH, Grumbach K. Improving primary care for patients with chronic illness: the chronic care model, part 2. *JAMA* 2002; 288: 1909–14. doi:10.1001/jama.288.15.1909
20. Campbell S M, Hann M, Hacker J, et al. Identifying predictors of high quality care in English general practice: observational study. *BMJ* 2001;323:784. doi:10.1136/bmj.323.7316.784
21. Tammes, P, Payne, RA, Salisbury, C, et al. The impact of a named GP scheme on continuity of care and emergency hospital admission: A cohort study among older patients in England, 2012-2016. *BMJ Open* 2019;vol.9,no.9,e029103. doi:10.1136/bmjopen-2019-029103
22. Arksey H, O'Malley L. Scoping studies: Towards a Methodological Framework. *Int J Soc Res* 2005;8:1,19-32. doi:10.1080/1364557032000119616
23. Tricco AC, et al. PRISMA Extension for Scoping Reviews (PRISMA-ScR): Checklist and Explanation. *Ann Intern Med* 2018 Oct 2;169(7):467-473. doi:10.7326/M18-0850
24. Peters, MDJ, Godfrey, C, Kahlil, H, et al. Guidance for conducting systematic scoping reviews. *Int J Evid Based Healthc* 2015;vol.13,ed.3,pp.141-46. doi:10.1097/XEB.0000000000000050
25. Levac D, Colquhoun H, O'Brien KK. Scoping studies: advancing the methodology. *Implement Sci* 2010;5(1):1. doi: 10.1186/1748-5908-5-6
26. Huxtable, R., Ives, J. Mapping, framing, shaping: a framework for empirical bioethics research projects. *BMC Med Ethics* 2019;20,86. doi:10.1186/s12910-019-0428-0
27. Ives, Dunn, Cribb. Empirical Bioethics: Theoretical and Practical Perspectives. Cambridge University Press 2016.
28. Hsieh HS, & Shannon S. Three Approaches to Qualitative Content Analysis. *Qualitative health research* 2005;15. 1277-88. doi:10.1177/1049732305276687

For peer review only

AUTHORS' CONTRIBUTIONS

SJO conceived the idea for the scoping review. SJO, JI and RH all contributed to development of the research questions and study design. SJO drafted the initial protocol with input from JI and RH. All authors contributed to re-drafting and subsequent final revision of this protocol. SJO is the guarantor of the review.

ACKNOWLEDGEMENTS

I would like to express my appreciation to Dr Giles Birchley, Dr Zuzana Deans and Dr Emanuele Valenti and Dr Iona Sherwood for the general advice, help and support they have given in conceiving this project.

FUNDING STATEMENT

This work was supported by the Elizabeth Blackwell Institute and Wellcome Trust Institutional Strategic Support Fund, grant number 204813/Z/16/Z. The Elizabeth Blackwell Institute and Wellcome Trust were not involved in the development of the protocol other than providing financial support.

COMPETING INTEREST STATEMENT

We have read and understood the BMJ policy on declaration of interests and declare that we have no competing interests.

WORD COUNT: 3041

For peer review only

Reporting checklist for protocol of a systematic review.

Based on the PRISMA-P guidelines.

Instructions to authors

Complete this checklist by entering the page numbers from your manuscript where readers will find each of the items listed below.

Your article may not currently address all the items on the checklist. Please modify your text to include the missing information. If you are certain that an item does not apply, please write "n/a" and provide a short explanation.

Upload your completed checklist as an extra file when you submit to a journal.

In your methods section, say that you used the PRISMA-Preporting guidelines, and cite them as:

Moher D, Shamseer L, Clarke M, Ghersi D, Liberati A, Petticrew M, Shekelle P, Stewart LA. Preferred Reporting Items for Systematic Review and Meta-Analysis Protocols (PRISMA-P) 2015 statement. Syst Rev. 2015;4(1):1.

		Reporting Item	Page Number
**Title**			
Identification	#1a	Identify the report as a protocol of a systematic review	1
Update	#1b	If the protocol is for an update of a previous systematic review, identify as such	n/a
**Registration**			
	#2	If registered, provide the name of the registry (such as PROSPERO) and registration number	n/a
**Authors**			
Contact	#3a	Provide name, institutional affiliation, e-mail address of all protocol authors; provide physical mailing address of corresponding author	1
Contribution	#3b	Describe contributions of protocol authors and identify the guarantor of the review	9
**Amendments**			
	#4	If the protocol represents an amendment of a previously completed or published protocol, identify as such and list changes; otherwise, state plan for documenting important protocol amendments	n/a
**Support**			

For peer review only

Sources	#5a	Indicate sources of financial or other support for the review	9
Sponsor	#5b	Provide name for the review funder and / or sponsor	9
Role of sponsor or funder	#5c	Describe roles of funder(s), sponsor(s), and / or institution(s), if any, in developing the protocol	n/a
**Introduction**			
Rationale	#6	Describe the rationale for the review in the context of what is already known	4
Objectives	#7	Provide an explicit statement of the question(s) the review will address with reference to participants, interventions, comparators, and outcomes (PICO)	4
**Methods**			
Eligibility criteria	#8	Specify the study characteristics (such as PICO, study design, setting, time frame) and report characteristics (such as years considered, language, publication status) to be used as criteria for eligibility for the review	5
Information sources	#9	Describe all intended information sources (such as electronic databases, contact with study authors, trial registers or other grey literature sources) with planned dates of coverage	6
Search strategy	#10	Present draft of search strategy to be used for at least one electronic database, including planned limits, such that it could be repeated	5
Study records - data management	#11a	Describe the mechanism(s) that will be used to manage records and data throughout the review	7
Study records - selection process	#11b	State the process that will be used for selecting studies (such as two independent reviewers) through each phase of the review (that is, screening, eligibility and inclusion in meta-analysis)	6
Study records - data collection process	#11c	Describe planned method of extracting data from reports (such as piloting forms, done independently, in duplicate), any processes for obtaining and confirming data from investigators	7
Data items	#12	List and define all variables for which data will be sought (such as PICO items, funding sources), any pre-planned data assumptions and simplifications	6
Outcomes and prioritization	#13	List and define all outcomes for which data will be sought, including prioritization of main and additional outcomes, with rationale	7
Risk of bias in individual studies	#14	Describe anticipated methods for assessing risk of bias of individual studies, including whether this will be done at the outcome or study level, or both; state how this information	n/a

For peer review only

		will be used in data synthesis	
Data synthesis	#15a	Describe criteria under which study data will be quantitatively synthesised	7
Data synthesis	#15b	If data are appropriate for quantitative synthesis, describe planned summary measures, methods of handling data and methods of combining data from studies, including any planned exploration of consistency (such as I2, Kendall's τ)	7
Data synthesis	#15c	Describe any proposed additional analyses (such as sensitivity or subgroup analyses, meta-regression)	7
Data synthesis	#15d	If quantitative synthesis is not appropriate, describe the type of summary planned	n/a
Meta-bias(es)	#16	Specify any planned assessment of meta-bias(es) (such as publication bias across studies, selective reporting within studies)	n/a
Confidence in cumulative evidence	#17	Describe how the strength of the body of evidence will be assessed (such as GRADE)	n/a

The PRISMA-P checklist is distributed under the terms of the Creative Commons Attribution License CC-BY 4.0. This checklist was completed on 25. February 2020 using https://www.goodreports.org/, a tool made by the EQUATOR Network in collaboration with Penelope.ai

For peer review only
